# Front-line clinical pharmacy

**DOI:** 10.1186/1757-7241-20-S2-P24

**Published:** 2012-04-16

**Authors:** Majken Nørskov Petersen, Marianne Brøndum Jensen, Anita Press, Peter Berlac, Dorthe Vilstrup Tomsen

**Affiliations:** 1 Capital Region of Denmark, Pharmacy, Pharmacy Unit North, Hillerød Hospital, Denmark; 2 Department of Emergency Medicine, Hillerød Hospital, Denmark

## Background

There is strong evidence supporting that pharmacist involvement strengthens the quality of the medication process. There is also evidence indicating that many emergency admissions among the elderly are medicine related. Based on this existing knowledge, it was decided to investigate whether implementation of clinical pharmacy in the Emergency Department could make a further difference.

## Methods

The Hillerød Hospital Emergency Department was chosen as case department for an implementation study, using "Model for Improvement" methodology.

Patients 50 years or older with at least five medications were included in the study.

The aim of the study was to investigate whether introduction of front-line clinical pharmacy could improve quality of medication in the Emergency Department and reduce medication related re-admissions.

## Results

A close collaboration between pharmacy managers and clinicians formed a necessary basis for coordinating and evaluating the combined efforts of medical, nursing and pharmaceutical staff. Clinicians pinpointed barriers and opportunities. Clinical and pharmacy managers accepted or rejected proposals and facilitated new workflows. Pharmacists strive to produce a valid medication status before clinicians see the patients.

Originally, only patients 65 years or older were to be included in the study. A pilot study on patients 50 years or older using 5 medications or more revealed, however, several patients between 50 and 65 years with drug related problems.

Validating medication history, prescriptions and current medication as well as "which pills are in the patient's handbag" has proved to be of critical value for clinicians. A well-defined workflow for front-line clinical pharmacy is developed.

Pharmacists document problem-oriented findings and recommendations in the patient record as well as alerting clinicians directly in more emergent cases.

The degree of clinicians taking action on pharmacists’ recommendations has increased from an initial 40 % to 80 % and still increasing.

Task acquisition from physicians to pharmacists has a tendency to reduced adverse events in connection with incorrect data entry into the hospital electronic prescribing system.

## Conclusion

The main preliminary finding of this study is the successful implementation of front-line clinical pharmacists in the Hillerød Hospital Emergency Department. This study has gathered invaluable knowledge regarding potential future implementation of clinical pharmacy in other emergency settings.

